# Alternative salt bridge formation in Aβ—a hallmark of early-onset Alzheimer's disease?

**DOI:** 10.3389/fmolb.2015.00014

**Published:** 2015-04-28

**Authors:** Maarten Schledorn, Beat H. Meier, Anja Böckmann

**Affiliations:** ^1^Physical Chemistry, Eidgenössische Technische Hochschule ZürichZurich, Switzerland; ^2^Institut de Biologie et Chimie des Protéines, Bases Moléculaires et Structurales des Systèmes Infectieux, Labex Ecofect, UMR 5086 CNRS, Université de LyonLyon, France

**Keywords:** amyloid-beta, Osaka mutant, Alzheimer's disease, 3D structure, solid-state NMR

## Abstract

Recently the 3D structure of the Osaka mutant form (E22Δ) of Amyloid-β1-40 has been determined. We here compare the NMR chemical-shift with the published shifts of a brain-seeded form of wild-type Aβ and suggest that the determined mutant fold is accessible to the wild-type protein as well, with small conformational adaptations which accommodate the E22 residue missing in the Osaka mutant. In addition, we illustrate how other mutants could also conform to this model. The stabilization of the N-terminal part of the protein via an intermolecular salt bridge to Lys28 may represent a common structural motif for the mutants which are related to early-onset Alzheimer disease. This feature might connect to the observed increased toxicity of the mutant forms compared to wild-type Aβ1-40, where the salt bridge involving Lys28 is intramolecular.

## Introduction

Amyloid-β (Aβ) in its different conformations and aggregation states is a central player in the amyloid-cascade hypothesis for Alzheimer's disease (AD) (Hardy and Higgins, 1992). Brain-derived or synthetic aggregates were shown to propagate between cells when injected into transgenic mice (Kane et al., 2000; Eisele et al., 2010). It is not established whether the Aβ fibrils or smaller oligomers, or both, are the toxic species (Benilova et al., 2012; Cohen et al., 2013; Matsuzaki, 2014). The existence of mutants that lead to early onset AD (Wu et al., 2012) offers an opportunity to characterize the conformational space available to the Aβ peptide in fibrillar or oligomeric state. Amyloids in general, and Aβ in particular, are known for their ability to form a number of polymorphs (Meier and Böckmann, 2015), and it has been proposed that they are at the origin of different phenotypes of the disease, reminiscent of the appearance of strains in prion disease (Meyer-Luehmann et al., 2006; Colby and Prusiner, 2011; Stöhr et al., 2012). Different mutant forms of Aβ have been identified involving a single-residue deletion or substitution at or adjacent to residue E22, e.g., Flemish A21G (Hendriks et al., 1992), Arctic E22G (Kamino et al., 1992; Nilsberth et al., 2001), Dutch E22Q (Levy et al., 1990; Van Broeckhoven et al., 1990), Italian E22K (Tagliavini et al., 1999; Bugiani et al., 2010), Iowa D23N (Grabowski et al., 2001), and the Osaka deletion mutation E22Δ (Tomiyama et al., 2008; Ovchinnikova et al., 2011). These mutants cause early-onset AD, and they display different toxicity profiles in *in vitro* and *in vivo* studies compared to wild-type Aβ1-40 and Aβ1-42.

Knowledge of the atomic-resolution 3D structure is central for the understanding of the molecular basis underlying the amyloid diseases, and solid-state NMR is a powerful method to determine and characterize structures of amyloid fibrils at atomic resolution (Wasmer et al., 2008) and to map the conformational space available to these proteins. In addition to the Osaka mutant structure, (Schütz et al., 2015) several wild-type Aβ fibril polymorphs have been characterized (Petkova et al., 2002; Paravastu et al., 2008; Bertini et al., 2011; Lopez del Amo et al., 2012; Lu et al., 2013; Niu et al., 2014), and a model for the Iowa mutant was presented (Sgourakis et al., 2015).

Despite a large body of literature, there is presently not enough high-resolution structural data available to establish a detailed structure-toxicity relationship for amyloids in general, and Aβ in particular (Tiller and Tessier, 2013). However, virtually complete chemical-shift information for all 40 residues has recently become available for two wild-type and a mutant form of Aβ1-40. We use this information here to suggest, on the basis of a comparison of NMR chemical-shift values between the Osaka mutant (Huber et al., 2014; Schütz et al., 2015) and one of the wild-type polymorphs (Lu et al., 2013), that the wild-type peptide has indeed the ability to assume the fold established for Aβ1-40 E22Δ (Schütz et al., 2015), albeit with a modified in-out pattern of the amino-acid residues in the loop comprising residues 20–31. Furthermore, we illustrate how other early-onset Aβ mutants can in principle form a similar fold. We speculate that the key feature of the mutant folds is the formation of an *inter*molecular salt bridge, attaching the N-terminal residues to the fibril core, as opposed to the wild-type protein, where the situation is substantially different, as residue K28 was experimentally shown to be involved in an *intra*molecular salt bridge (Lu et al., 2013). As a consequence, the N-terminus is less tightly attached, which impacts fibril properties, and potentially those of prefibrillar states (Tarus et al., 2006; Reddy et al., 2009).

## Materials and methods

### Calculation of the model for the WT Aβ dimer

**Figure 1:**
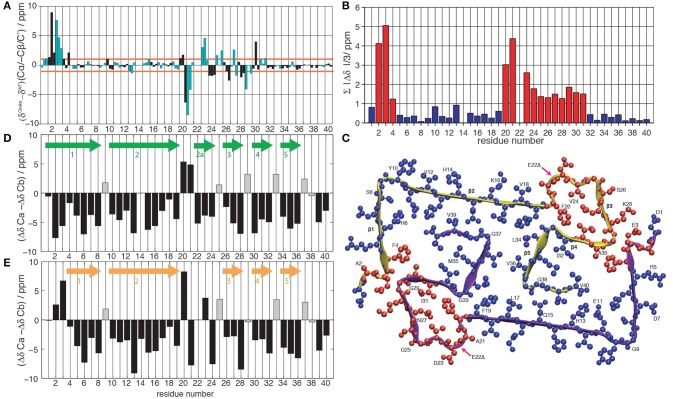
**(A)** Difference between the chemical shifts Cα, -Cβ and C' between the Osaka mutant (Schütz et al., 2015) and wild-type Aβ 1-40, Patient I of Lu et al. (2013). Cβ is given with negative values to highlight differences in secondary structure propensity where all chemical-shift differences point in the same direction; where the Osaka mutant shows less β-strand structure, all three values are positive, and vice versa. The chemical shifts are re-referenced to yield the smallest possible differences. Even residues are shown in black, odd ones in cyan. **(B)** Average absolute values of the mean chemical shift differences between wild-type Aβ 1-40 (Lu et al., 2013) and the Osaka mutant (Schütz et al., 2015), $(\left|\delta C\alpha^{WT}-\delta C\alpha^{Osaka}\right|+\left|\delta C\beta^{WT}-\delta C\beta^{Osaka}\right|+\left|\delta {C^{\prime }}^{WT}-\delta {C^{\prime }}^{Osaka}\right|)/3$. Deviations larger than 1 ppm are plotted in red, those smaller in blue. **(C)** Representation of chemical-shift differences from **(B)** plotted on the Aβ 1-40 E22Δ structure (pdb entry 2MVX). Same color coding as in **(B)**. Differences between Cα and Cβ secondary chemical shifts for **(D)** wild-type Aβ 1-40 Patient I of Lu et al. (2013) and **(E)** the Osaka mutant (Schütz et al., 2015). Arrows indicate locations of β-sheets according to the rule that three negative values in a row are indicative for this secondary structure element, with the exception of glycines which are marked by a gray bar color.

The calculation was performed as described for the “manual calculations” in reference (Schütz et al., 2015). For residues 2–4, and 20–31, TALOS+ angles were calculated from the deposited chemical shifts (Lu et al., 2013). Unambiguous distance restraints, as determined in Schütz et al. (2015) for the mutant form, were used for the residues which show concomitant chemical shifts in both proteins (blue residues in Figure 1C). The list of distance restraints used is therefore a subset of the list given in Schütz et al. (2015), namely the ones between two residues from the range 1, 5–19, 32–40, and is listed in Table S1. The orientations of the β-strands were defined for β-sheets 1, 2, 4, and 5 in the same manner as in the mutant form (for the strand numbering see Figures 1D–E). This input was not sufficient to distinguish between the possible orientations of the two β-strands spanning residues E22-V24 (strand 2a) and S26-K28 (strand 3) in the wild-type protein (Figure 1D) on the basis of restraint violations. The following additional considerations were used as constraints for the structure calculations: (i) The assigned side-chain Cγ/δ chemical shifts in the brain-seeded WT polymorph for D23 and E22 respectively show chemical-shift values typical of *charged* COO^−^ side-chain moieties (Lu et al., 2013). (ii) The β-sheet structure of E22-V24 evidenced by the secondary chemical shifts (Figure 1D) observed for the brain-seeded WT polymorph implies that either E22 or D23 point to the inside of the fibril. (iii) The thus introduced E22 or D23 negative charge inside the fibril should be compensated by a positive charge in order to yield a stable fibril. Here, K28 is the only possible partner. From these three points, we can infer that S26-K28 shows an in-out-in pattern, allowing for charge compensation. This leaves the two options of an out-in-out or in-out-in orientation for E22-V24, but steric clashes between V24 and S26 (in an in-out-in orientation of E22-V24) strongly suggest an out-in-out pattern, with D23 pointing inside. And indeed, the D23-K28 salt bridge has been described in most WT models (Paravastu et al., 2006; Lu et al., 2013).

### Structural models for the mutants

**Figure 2:**
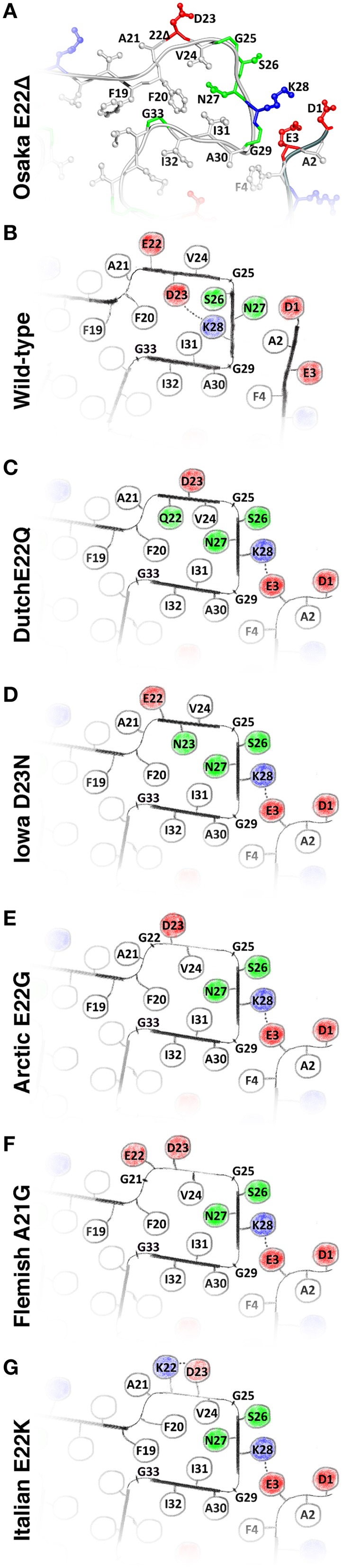
**(A)** The structure of the Osaka mutant E22Δ from Schütz et al. (2015); **(B)** speculative model of WT Aβ as described in the main text; and hypothetical models of **(C)** Dutch mutant E22Q; **(D)** Iowa mutant D23N; **(E)** Arctic mutant E22G; **(F)**; Flemish mutant A21G; and **(G)** Italian mutant E22K. Models **(C–G)** are built based on the Osaka mutant structure and additional considerations as described in the main text. All these mutants favor, in this model, an outside orientation of K28. Hydrophobic residues are colored white, glycines and polar residues are green, and charged residues are colored blue when positive and red when negative.

For all mutant form models, dihedral angle restraints from Schütz et al. (2015) were used and distance restraints from the same source were entered into CYANA calculations, except for residues 20–30 and 2–4. Hydrogen bonding patterns were adopted from the Osaka mutant for the Arctic, Flemish and Italian mutant. Like the WT model, the Dutch and Iowa mutant calculations included an additional β-sheet 2a for residues 22–24 (see Figure 1D). Its orientation followed from two arguments: (i) we avoid uncompensated charges inside the core of the fibril, and (ii) we avoid steric clashes between residues V24 and S26. As a result, the Iowa mutant has an out-in-out pattern (Figure 2D and Figure S2C), as also predicted in a recent model for the Iowa mutant (Sgourakis et al., 2015). While the Dutch mutant assumes an in-out-in pattern for residues Q22-V24 (Figure 2C and Figure S2B), the Arctic mutant shows an outside-pointing D23 (Figure 2E and Figure S2D), and in the Flemish mutant both E22 and D23 are oriented outwards and β-strand E22-V24 is not present (Figure 2F and Figure S2E). Finally, in the Italian mutant (Figure 2G and Figure S2F), we force the formation of a K22-D23 salt bridge by adding a restraint to the CYANA where no electrostatic interactions are considered. Without restraint violations, this residue pair can only be accommodated outside the loop due to limited space on the inside. The structural statistics for all mutant calculations as well as the wildtype is given in Table S2.

## Results and discussion

### The wild-type protein can adopt a similar fold as the osaka mutant

Chemical-shift comparisons between proteins represent a sensitive means to assess differences and similarities in 3D structures, as shift differences point to conformational differences for the observed residue. Comparison of wild-type Aβ chemical shifts described by Lu et al. (2013) to those of the Aβ1-40 E22Δ mutant (Schütz et al., 2015) (Figure 1A) reveals that the two forms display almost coincident shifts for large parts of the protein, with noticeable exceptions in two regions where the differences surpass the 1 ppm limit which we consider to be a significant change.

To illustrate the location of these changes, we plotted the absolute value of the mean chemical-shift differences from Figure 1A, displayed in Figure 1B, on the Aβ E22Δ mutant structure in Figure 1C: residues with similar shifts are colored in blue, others in red. Blue residues cover the entire hydrophobic core, as well as the largest parts of β-sheets 1 and 2. Importantly, the two regions displaying larger chemical-shift differences are distant in sequence, but spatially contiguous if plotted on the Aβ E22Δ mutant structure. They concern the loop as well as the N-terminus attached to the loop via the E3-K28 salt bridge. For comparison, the other wild-type polymorph with complete assignments (Bertini et al., 2011) shows substantial chemical-shift differences spanning the entire sequence, with only few and possibly fortuitous exceptions (see Supplementary Figures S1A–C).

### Comparison of secondary structural features of WT Aβ and the osaka mutant

Secondary chemical shifts [the difference to random-coil shifts (Bax, 1991; Wishart and Sykes, 1994)] inform on protein secondary structures. The secondary shift differences ΔδCα−ΔδCβ can be evaluated according to a pattern of three consecutive negative values corresponding to a β-strand, four positive values in a row indicating α-helical secondary structure, and the remainder turns or loops. ΔδCα−ΔδCβ of Aβ1-40 and Aβ1-40 E22Δ is plotted in Figures 1D,E and the resulting pattern of β-sheets is found to be quite similar, with a couple of noticeable differences: for residues A2 and G3, where the mutant protein structure presents a turn, the WT protein chemical shifts indicate a straight β-strand, extending the strand which in Aβ1-40 E22Δ spans residues F4 to S8. The turn comprising residues F20-V24 in the mutant form is reduced, in the WT peptide, to residues F20-A21, and an additional β-strand, E22-V24 is present. We note from Figure 1 that the β-sheet S26-K28 is conserved, but that the individual chemical shifts (Figure 1A) differ significantly (>1 ppm). Residues F4–F19 and residues I30-V40 feature very similar shifts for the WT and the Osaka-mutant fibril.

### A speculative structure for a WT Aβ polymorph

We have noted that the monomer conformation for the residues mentioned above is very similar for the WT from the brain-seeded sample and the Osaka mutation despite the fact that the polymorph of the mutant form is described by a two-fold symmetry (Schütz et al., 2015) and the WT polymorph by a three-fold symmetry (Lu et al., 2013). In the following, we construct a two-fold model of a monomer in a WT fibril using the information provided in Table 1. The model mainly relies on the data coming from the mutant form, complemented by information from the brain seeded WT form for the residues showing chemical shift differences between the two forms. Details are given in the Materials and Methods Section. The corresponding calculation resulted in no restraint violations, and the atomic-detail model is displayed for information in Figure S2. Figure 2B represents the region of interest as a schematic drawing, in order to emphasize its nature as a speculative model. When compared to the Osaka mutant structure shown in Figure 2A, the two β-sheets E22-V24 and S26-K28 in this model are turned around (inside/outside interchanged) to accommodate the K28-D23 salt bridge inside the loop (see Materials and Methods Section). The attachment of β-sheet 1 (Figure 1C) is now only mediated by the H6-E11-E13-V40 salt bridge network (Schütz et al., 2015), as well as F4-A30 hydrophobic interactions, and lacks the strong electrostatic stabilization by E3-K28. This would explain the dynamic nature of the N-terminal part, roughly 10 residues, which has systematically been observed in the WT forms. These residues were shown to display a certain flexibility (allowing for H/D exchange or giving raise to weak or missing signals in solid-state NMR spectra) in most polymorphs described, and could only be sequentially assigned for Aβ1-40 E22Δ and three wild-type models (Bertini et al., 2011; Lu et al., 2013; Niu et al., 2014). Even in these, no unambiguous distance restraints could be established between the N-terminal segment and the fibril core. Only the Aβ1-40 E22Δ mutant distinguishes itself by a rigid N-terminal segment, structurally unambiguously defined by the intermolecular E3-K28 salt bridge. We speculate that this stabilizing salt bridge is a direct consequence of the E22Δ mutation, and we illustrate in the following how it might occur as well in other early-onset AD related Aβ variants, where mutations are found in the in the loop around residue A21-D23.

**Table 1:** **Information used as input for WT model building**.

	**Residues 5–19 and 32–40**		**Residues 1–4 and 20–31**	
Distance restraints	Inter/Intra-molecularly and spectrally unambiguous restraints		None	
TALOS+ angles	BMRB 25289 (Schütz et al., 2015)		BMRB 19009 (Lu et al., 2013)	
Sequence	1	10	20	30
	DAEFRHDSG	YEVHHQKLVF	FAEDVGSNKG	AIIGLMVGGVV
β-Strands	1----	2---------	2a- 3--	4-- 5--
Orientation of first residue	In	Out	Out In	Out In

*Distance restraints from the Osaka mutant were used between the residues which show small chemical shift-differences (blue in Figure 1C). A list is given in Table S1. TALOS+ angles were calculated from the given chemical shift depositions and the restraints for CYANA calculations had a range over the top 10 database matches ± 15°. Residues without dihedral angle restraints were: all glycines; residues D1 and V40 being the terminal residues; and residues A21 and V24 which had a warning in the TALOS+ prediction. Although F20 also has a warning in the TALOS+ prediction, the restraint was still used, since secondary chemical shifts in both the WT (Lu et al., 2013) and the Osaka mutant (Schütz et al., 2015) convincingly indicate that the β-strand that starts from Y10 ends at F19. F20 thus should discontinue the strand and have the same orientation as F19. β-strands were established according to Figure 3 and Figure S8 in Schütz et al. (2015). Strand 2a does not exist in the Osaka mutant, but is suggested in the WT form by the chemical shifts (Lu et al., 2013) (Figure 1D). The orientation of the side chains is defined by the hydrogen bonding pattern between the different layers of the fibril and the resultant orientation of the first residue in each strand is given as inward or outward with respect to the core of the dimer*.

### Structural models for other mutant forms

A common feature shared by all the here discussed early-onset mutants is that their amino-acids composition is such that the presence of a negative charge in the fibril interior can be avoided, which in turn allows an outside orientation of Lys28 and a rigidification of the N-terminal β-strand by its attachment to the fibril core via the E3-K28 salt bridge. Hypothetical models for the different mutant forms are shown in Figure S2 (for details of the calculations, see Materials and Methods Section), and drawings of the regions of interest are shown in Figures 2C–G. The Dutch, Iowa and Arctic mutants all result in the replacement of a negative charge of the wild-type peptide, either D23 or E22, by a polar residue, which easily accommodates then a charge-free fibril interior in the loop encompassing residues 20–31. In the Flemish mutant, the A21G mutation introduced additional flexibility, which can accommodate an outside orientation of both E22 and D23. In the Italian mutant, replacement of E22 by a positive charge (K22) allows to stabilize D23 outside by a salt bridge with K22. The calculations carried out for the mutant forms (Figure S2) show that the above discussed hypothetical conformations can be realized without steric hindrance.

## Conclusion

In summary, while we cannot ascertain from our calculations that Lys28 must be pointing outside in the early onset Aβ mutants discussed here, we could show that there are no restraint violations, static clashes or unbalanced charges inside the loop when Lys28 points outside. Consequently, the residue can engage in an energetically favorable salt bridge with the N-terminus of the protein. The Osaka polymorph fold is thus in principle accessible to the other mutant forms, and the possibility to assume this fold could be of importance to early onset. In any case, the models presented here provide presently testable hypotheses in mutational studies allowing to experimentally confirm the central role of K28 in the increased toxicity of mutant forms (Levy et al., 1990; Van Broeckhoven et al., 1990; Hendriks et al., 1992; Tagliavini et al., 1999; Grabowski et al., 2001; Nilsberth et al., 2001; Tomiyama et al., 2008; Bugiani et al., 2010). More chemical-shift information and high-resolution structures of different polymorphs of WT Aβ and mutant fibrils of both Aβ1-40 and 1-42 and, hopefully, oligomers (Stroud et al., 2012), will be needed to reliably establish a clear relationship between structure and biological activity of the protein.

### Conflict of interest statement

The authors declare that the research was conducted in the absence of any commercial or financial relationships that could be construed as a potential conflict of interest.

## References

[B1] Bax A. (1991). Empirical correlation between protein backbone conformation and C-alpha and C-beta C-13 nuclear-magnetic-resonance chemical-shifts. J. Am. Chem. Soc. 113, 5490–5492.

[B2] Benilova I. Karran E. De Strooper B. (2012). The toxic Aβ oligomer and Alzheimer's disease: an emperor in need of clothes. Nat. Publ. Group 15, 349–357. 10.1038/nn.3028 22286176

[B3] Bertini I. Gonnelli L. Luchinat C. Mao J. Nesi A. (2011). A new structural model of Aβ 40 fibrils. J. Am. Chem. Soc. 133, 16013–16022. 10.1021/ja2035859 21882806

[B4] Bugiani O. Giaccone G. Rossi G. Mangieri M. Capobianco R. Morbin M.. (2010). Hereditary cerebral hemorrhage with amyloidosis associated with the E693K mutation of APP. Arch. Neurol Chic. 67, 987–995. 10.1001/archneurol.2010.178 20697050

[B5] Cohen S. I. A. Linse S. Luheshi L. M. Hellstrand E. White D. A. Rajah L.. (2013). Proliferation of amyloid-β 42 aggregates occurs through a secondary nucleation mechanism. Proc. Natl. Acad. Sci. U.S.A. 110, 9758–9763. 10.1073/pnas.1218402110 23703910 PMC3683769

[B6] Colby D. W. Prusiner S. B. (2011). Prions. Cold Spring Harb. Perspect. Biol. 3:a006833. 10.1101/cshperspect.a006833 21421910 PMC3003464

[B7] Eisele Y. S. Obermueller U. Heilbronner G. Baumann F. Kaeser S. A. Wolburg H.. (2010). Peripherally applied a beta-containing inoculates induce cerebral beta-amyloidosis. Science 330, 980–982. 10.1126/science.1194516 20966215 PMC3233904

[B8] Grabowski T. J. Cho H. S. Vonsattel J. P. Rebeck G. W. Greenberg S. M. (2001). Novel amyloid precursor protein mutation in an Iowa family with dementia and severe cerebral amyloid angiopathy. Ann. Neurol. 49, 697–705. 10.1002/ana.1009 11409420

[B9] Hardy J. Higgins G. (1992). Alzheimer's disease: the amyloid cascade hypothesis. Science 256, 184–185. 10.1126/science.1566067 1566067

[B10] Hendriks L. van Duijn C. Cras P. Cruts M. van Hul W. van Harskamp F.. (1992). Presenile dementia and cerebral haemorrhage linked to a mutation at codon 692 of the β-amyloid precursor protein gene. Nat. Genet. 1, 218–221. 1303239 10.1038/ng0692-218

[B11] Huber M. Ovchinnikova O. Y. Schütz A. K. Glockshuber R. Meier B. H. Böckmann A. (2014). Solid-state NMR sequential assignment of Osaka-mutant amyloid-beta (Aβ 1-40 E22Δ) fibrils. Biomol. NMR Assign. 9, 7–14. 10.1007/s12104-013-9535-x 24395155

[B12] Kamino K. Orr H. T. Payami H. Wijsman E. M. Alonso M. E. Pulst S. M.. (1992). Linkage and mutational analysis of familial Alzheimer disease kindreds for the APP gene region. Am. J. Hum. Genet. 51, 998–1014. 1415269 PMC1682859

[B13] Kane M. D. Lipinski W. J. Callahan M. J. Bian F. Durham R. A. Schwarz R. D.. (2000). Evidence for seeding of beta-amyloid by intracerebral infusion of Alzheimer brain extracts in beta-amyloid precursor protein-transgenic mice. J. Neurosci. 20, 3606–3611. 10804202 10.1523/JNEUROSCI.20-10-03606.2000 PMC6772682

[B14] Levy E. Carman M. D. Fernandez-Madrid I. J. Power M. D. Lieberburg I. van Duinen S. G.. (1990). Mutation of the Alzheimer's disease amyloid gene in hereditary cerebral hemorrhage, Dutch type. Science 248, 1124–1126. 10.1126/science.2111584 2111584

[B15] Lopez del Amo J.-M. Schmidt M. Fink U. Dasari M. Fändrich M. Reif B. (2012). An asymmetric dimer as the basic subunit in Alzheimer's disease amyloid β fibrils. Angew. Chem. Int. Ed Engl. 51, 6136–6139. 10.1002/anie.201200965 22565601

[B17] Lu J.-X. Qiang W. Yau W.-M. Schwieters C. D. Meredith S. C. Tycko R. (2013). Molecular structure of beta-amyloid fibrils in Alzheimer's disease brain tissue. Cell 154, 1257–1268. 10.1016/j.cell.2013.08.035 24034249 PMC3814033

[B18] Matsuzaki K. (2014). How do membranes initiate Alzheimer's Disease? formation of toxic amyloid fibrils by the amyloid β-protein on ganglioside clusters. Acc. Chem. Res. 47, 2397–2404. 10.1021/ar500127z 25029558

[B19] Meier B. H. Böckmann A. (2015). ScienceDirectThe structure of fibrils from “misfolded” proteins. Curr. Opin. Struct. Biol. 30, 43–49 10.1016/j.sbi.2014.12.001 25544255

[B20] Meyer-Luehmann M. Coomaraswamy J. Bolmont T. Kaeser S. Schaefer C. Kilger E.. (2006). Exogenous induction of cerebral beta-amyloidogenesis is governed by agent and host. Science 313, 1781–1784. 10.1126/science.1131864 16990547

[B21] Nilsberth C. Westlind-Danielsson A. Eckman C. B. Condron M. M. Axelman K. Forsell C.. (2001). The ‘Arctic’APP mutation (E693G) causes Alzheimer's disease by enhanced A[beta] protofibril formation. Nat. Neurosci. 4, 887–893. 10.1038/nn0901-887 11528419

[B22] Niu Z. Zhao W. Zhang Z. Xiao F. (2014). The molecular structure of Alzheimer β−amyloid fibrils formed in the presence of phospholipid vesicles. Angew. Chemie 35, 9294–9297. 10.1002/anie.201311106 24810551

[B23] Ovchinnikova O. Y. Finder V. H. Vodopivec I. Nitsch R. M. Glockshuber R. (2011). The Osaka FAD mutation E22Δ leads to the formation of a previously unknown type of amyloid β fibrils and modulates Aβ neurotoxicity. J. Mol. Biol. 408, 780–791. 10.1016/j.jmb.2011.02.049 21402079

[B24] Paravastu A. K. Leapman R. D. Yau W.-M. Tycko R. (2008). Molecular structural basis for polymorphism in Alzheimer's beta-amyloid fibrils. Proc. Natl. Acad. Sci. U.S.A. 105, 18349–18354. 10.1073/pnas.0806270105 19015532 PMC2587602

[B25] Paravastu A. Petkova A. Tycko R. (2006). Polymorphic fibril formation by residues 10-40 of the Alzheimer's {beta}-amyloid peptide. Biophys. J. 90, 4618–4629. 10.1529/biophysj.105.076927 16565054 PMC1471876

[B26] Petkova A. Ishii Y. Balbach J. Antzutkin O. Leapman R. Delaglio F.. (2002). A structural model for Alzheimer's {beta}-amyloid fibrils based on experimental constraints from solid state NMR. Proc. Natl. Acad. Sci. U.S.A. 99, 16742–16747. 10.1073/pnas.262663499 12481027 PMC139214

[B27] Reddy G. Straub J. E. Thirumalai D. (2009). Influence of preformed Asp23-Lys28 salt bridge on the conformational fluctuations of monomers and dimers of Aβ Peptides with implications for rates of fibril formation. J. Phys. Chem. B 113, 1162–1172. 10.1021/jp808914c 19125574 PMC3098509

[B28] Schütz A. K. Vagt T. Huber M. Ovchinnikova O. Y. Cadalbert R. Wall J.. (2015). Atomic-resolution three-dimensional structure of amyloid β fibrils bearing the osaka mutation. Angew. Chem. Int. Ed. Engl. 54, 331–335. 10.1002/anie.201408598 25395337 PMC4502972

[B29] Sgourakis N. G. Yau W.-M. Qiang W. (2015). Modeling an in-register, parallel “iowa” A&beta; fibril structure using solid-state NMR data from labeled samples with rosetta. Struct. Fold. Des. 23, 216–227. 10.1016/j.str.2014.10.022 25543257

[B30] Stöhr J. Watts J. C. Mensinger Z. L. Oehler A. Grillo S. K. DeArmond S. J.. (2012). Purified and synthetic Alzheimer's amyloid beta (Aβ) prions. Proc. Natl. Acad. Sci. U.S.A. 109, 11025–11030. 10.1073/pnas.1206555109 22711819 PMC3390876

[B31] Stroud J. C. Liu C. Teng P. K. Eisenberg D. (2012). Toxic fibrillar oligomers of amyloid-β have cross-β structure. Proc. Natl. Acad. Sci. U.S.A. 109, 7717–7722. 10.1073/pnas.1203193109 22547798 PMC3356606

[B32] Tagliavini F. Rossi G. Padovani A. Magoni M. Andora G. Sgarzi M. (1999). A new app mutation related to hereditary cerebral haemorrhage. Alzheimers Rep. 2, S28.

[B33] Tarus B. Straub J. E. Thirumalai D. (2006). Dynamics of Asp23-Lys28 salt-bridge formation in Aβ 10-35monomers. J. Am. Chem. Soc. 128, 16159–16168. 10.1021/ja064872y 17165769

[B34] Tiller K. E. Tessier P. M. (2013). Lifting the veil on amyloid drug design. eLife 2:e01089. 10.7554/eLife.01089 23878728 PMC3713451

[B35] Tomiyama T. Nagata T. Shimada H. Teraoka R. Fukushima A. Kanemitsu H.. (2008). A new amyloid β variant favoring oligomerization in Alzheimer's-type dementia. Ann. Neurol. 63, 377–387. 10.1002/ana.21321 18300294

[B36] Van Broeckhoven C. Haan J. Bakker E. Hardy J. A. van Hul W. Wehnert A.. (1990). Amyloid beta protein precursor gene and hereditary cerebral hemorrhage with amyloidosis (Dutch). Science 248, 1120–1122. 10.1126/science.1971458 1971458

[B37] Wasmer C. Lange A. van Melckebeke H. Siemer A. B. Riek R. Meier B. H. (2008). Amyloid fibrils of the HET-s(218-289) prion form a beta solenoid with a triangular hydrophobic core. Science 319, 1523–1526. 10.1126/science.1151839 18339938

[B38] Wishart D. S. Sykes B. D. (1994). The 13C chemical-shift index: a simple method for the identification of protein secondary structure using 13C chemical-shift data. J. Biomol. NMR 4, 171–180. 10.1007/BF00175245 8019132

[B39] Wu L. Rosa-Neto P. Hsiung G.-Y. R. Sadovnick A. D. Masellis M. Black S. E.. (2012). Early-onset familial Alzheimer's disease (EOFAD). Can. J. Neurol. Sci. 39, 436–445. 10.1017/S0317167100013949 22728850

