# Supplementary material for: Alternative salt bridge formation in Aβ—a hallmark of early-onset Alzheimer's disease?
Source: Front Mol Biosci. 2015 Apr 28;2:14. doi: 10.3389/fmolb.2015.00014 (PMC4429654; doi:10.3389/fmolb.2015.00014)
Supplement: Supplementary file 1 [file Presentation1.PDF]

Supplementary Material

Alternative salt bridge formation in Aβ – a hallmark of early-onset Alzheimer's disease?

Maarten Schledorn[1], **Beat H. Meier**[1]* & **Anja Böckmann**[2]*

[1]Physical Chemistry, ETH Zürich, Vladimir-Prelog-Weg 2, CH-8093 Zurich, Switzerland.
[2]Institut de Biologie et Chimie des Protéines, Bases Moléculaires et Structurales des Systèmes Infectieux, Labex Ecofect, UMR 5086 CNRS, Université de Lyon, 7 passage du Vercors, 69367 Lyon, France

*** Correspondence:** Anja Böckmann, IBCP CNRS, 7, passage du Vercors, 69367 Lyon Cedex 07, a.bockmann@ibcp.fr and Beat H. Meier, Physical Chemistry, ETH Zürich, Vladimir-Prelog-Weg 2, CH-8093 Zurich, Switzerland, beme@ethz.ch

1.1. Supplementary Figures

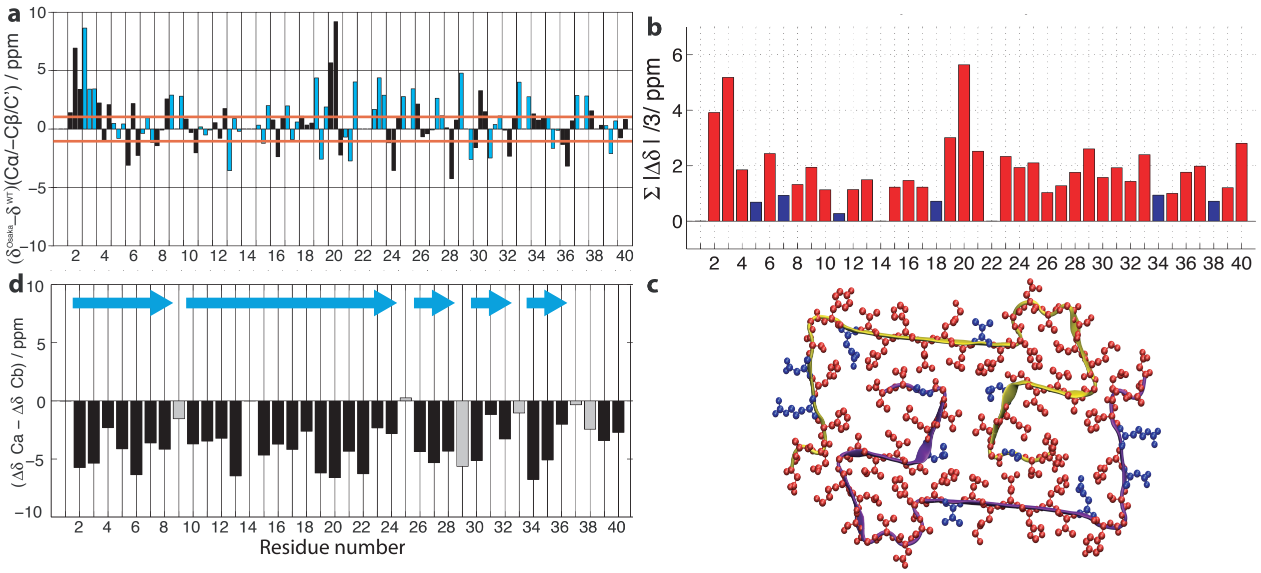

Supplementary Figure 1. (A) Difference between the chemical shifts Cα, -Cβ and C' between the Osaka mutant (Schütz et al., 2015) and the wild-type Aβ1-40 described by (Bertini et al., 2011). Cβ is given with negative values to highlight differences in secondary structure propensity where all chemical-shift differences point in the same direction; where the Osaka mutant shows less β-strand structure, all three values are positive, and vice versa. Even residues are shown in black, odd ones in cyan. (B) Absolute values of the mean chemical shift differences between wild-type Aβ 1-40 (Bertini et al., 2011) and the Osaka mutant (Schütz et al., 2015). Deviations larger than 1 ppm are plotted in red, those smaller in blue. (C) Representation of the chemical-shift differences between WT Aβ1-40 (Bertini et al., 2011) and Aβ1-40 E22Δ on a residue by residue basis plotted on the Aβ1-40 E22Δ

structure (pdb entry 2MVX) with the residues showing mean chemical-shift differences larger than 1 ppm in red, and smaller than 1 ppm in blue. (D) secondary chemical shifts for the polymorph described by (Bertini et al., 2011) suggesting five beta-strands, assuming that each glycine interrupts the strand even for a negative secondary Cα chemical shift (Wasmer et al., 2008). Indeed, G9 seems to interrupt the first long stretch, as no long-range restraints were detected for these residues and the structural model displays a manually added dynamic first β-sheet including residues 1-8 (Bertini et al., 2011). Glyine 25 clearly points to a turn, and, considering the resulting structural model (Bertini et al., 2011), Gly 29 must represent a second turn. However, unambiguous distance restraints have been detected neither for the N-terminal strand, nor for residues 22-30, for which the structure remains undefined.

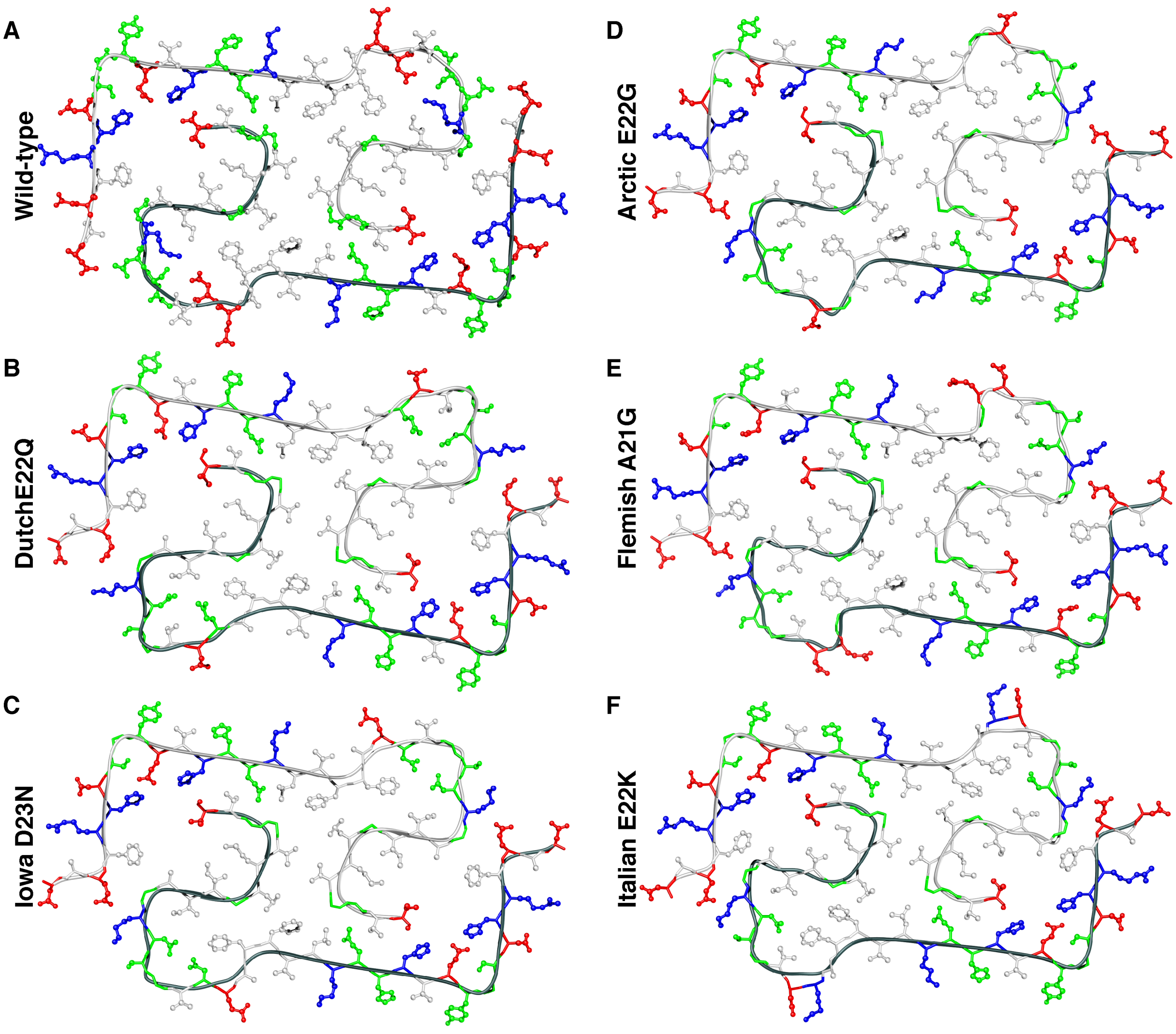

Supplementary Figure 2. Atomic-detail models that resulted from CYANA structure calculations and which form the basis for the hand-drawn pictures in Figure 2, which emphasizes the hypothetical nature of these models. Hydrophobic residues are colored white, glycines and polar residues are green, and charged residues are colored blue when positive and red when negative. The statistics for the structure calculations is given in Table S2.

1.2. Supplementary Tables

DARR	ω_1	ω_2
~~**E3-K28**~~	~~E3 CA~~	~~K28 CG~~
	~~K28 CG~~	~~E3 CA~~
	~~E3 CA~~	~~K28 CD~~
~~**E3-G29**~~	~~G29 C~~	~~E3 CA~~
~~**E3-A30**~~	~~E3 CA~~	~~A30 CA~~
	~~A30 CA~~	~~E3 CA~~
**H13-V40**	H13 CE1	V40 CA
	V40 CA	H13 CE1
	V40 C	H13 CA
	V40 C	H13 CD2
	V40 C	H13 CE1
	V40 CA	H13 CD2
**H6-E11**	H6 CD2	E11 CG
	E11 CG	H6 CE1
**F19-L34**	F19 CB	L34 CG
	L34 CG	F19 CA
	L34 CG	F19 CZ
~~**F20-I31**~~	~~F20 CG~~	~~I31 CG2~~
	~~F20 CE1~~	~~I31 CG2~~
**I32-M35**	I32 CG2	M35 CB
	M35 CB	I32 CG2
**M35-G38**	M35 CA	G38 C
	G38 C	M35 CA
	G38 C	M35 CE

PAR	ω_1	ω_2
~~**E3-K28**~~	~~E3 CA~~	~~K28 CG~~
	~~K28 CG~~	~~E3 CA~~
~~**E3-A30**~~	~~E3 CA~~	~~A30 CA~~
	~~A30 CA~~	~~E3 CA~~
**H6-H13**	H6 CE1	H13 CE1
	H13 CE1	H6 CE1
**F19-L34**	L34 CG	F19 CA
	L34 CG	F19 CB
**I32-M35**	I32 CG2	M35 CB
	M35 CB	I32 CG2
	I32 CD1	M35 CB
	M35 CB	I32 CD1

CHHC	ω_1	ω_2
~~**E3-K28**~~	~~E3 HA~~	~~K28 QG~~
**H6-H13**	H6 HE1	H13 HE1
	H13 HE1	H6 HE1
**F19-L34**	F19 QB	L34 HG
**I32-M35**	I32 QG2	M35 QB
	M35 QB	I32 QG2
	M35 QB	I32 QD1

PAIN	ω_1	ω_2
~~**E3-G29**~~	~~E3 CA~~	~~G29 N~~
**H13-V40**	V40 C	H13 ND1
	V40 CA	H13 ND1
**Q15-G37**	G37 CA	Q15 NE2
**Q15-G38**	G38 C	Q15 NE2

Supplementary Table 1. Unambiguous restraints extracted from Aβ1-40 E22Δ. The basic table (without the strikeouts) is reproduced from ref. (Schütz et al., 2015) (with permission). Restraints that are struck through were not used here for the CYANA manual calculations of WT and other mutants. A red background indicates intermolecular restraints, while intramolecular restraints have a gray background. For details on the restraint collection and the calculations, see reference (Schütz et al., 2015).

	Wild-type	Dutch E22Q	Iowa D23N	Arctic E22G	Flemish A21G	Italian E22K
**Target function**	$(9 \pm 4) \cdot 10^{-3}$	$(4.1 \pm 0.2) \cdot 10^{-2}$	$(2 \pm 3) \cdot 10^{-4}$	$(5 \pm 2) \cdot 10^{-5}$	$(3 \pm 1) \cdot 10^{-5}$	$(4 \pm 3) \cdot 10^{-4}$
**Violations (mean and s.d.)**						
Distance constraints (Å)	$(3 \pm 1) \cdot 10^{-4}$	$(2.0 \pm 0.1) \cdot 10^{-4}$	$< 10^{-5}$	$< 10^{-5}$	$< 10^{-5}$	$< 10^{-5}$
Dihedral angle constraints (°)	$(1.8 \pm 0.7) \cdot 10^{-2}$	$(2.1 \pm 0.1) \cdot 10^{-2}$	$(1 \pm 1) \cdot 10^{-3}$	$< 10^{-5}$	$< 10^{-5}$	$(2 \pm 1) \cdot 10^{-3}$
Max. distance constraint violation (Å)	$(1.0 \pm 0.1) \cdot 10^{-2}$	$< 10^{-5}$	$< 10^{-5}$	$< 10^{-5}$	$< 10^{-5}$	$< 10^{-5}$
Max. dihedral angle constraints (°)	$(1.4 \pm 0.5) \cdot 10^{-1}$	$(2.2 \pm 0.2) \cdot 10^{-1}$	$(1 \pm 1) \cdot 10^{-2}$	$< 10^{-5}$	$< 10^{-5}$	$(2 \pm 1) \cdot 10^{-2}$
**Average pairwise r.m.s.d.**						
Backbone (Å)	0.6 ± 0.2	0.4 ± 0.1	1.2 ± 0.4	1.0 ± 0.3	0.7 ± 0.1	1.1 ± 0.3
Heavy atom (Å)	0.9 ± 0.1	0.7 ± 0.1	1.5 ± 0.4	1.3 ± 0.3	0.9 ± 0.1	1.4 ± 0.3

Supplementary Table 2. CYANA statistics for the structural models presented in Supplementary Figure 2.

Bertini, I., Gonnelli, L., Luchinat, C., Mao, J., and Nesi, A. (2011). A New Structural Model of Aβ40 Fibrils. *J Am Chem Soc* 133, 16013–16022. doi:10.1021/ja2035859.

Lu, J.-X., Qiang, W., Yau, W.-M., Schwieters, C. D., Meredith, S. C., and Tycko, R. (2013b). Molecular Structure of beta-Amyloid Fibrils in Alzheimer's Disease Brain Tissue. *Cell* 154, 1257–1268. doi:10.1016/j.cell.2013.08.035.

Schütz, A. K., Vagt, T., Huber, M., Ovchinnikova, O. Y., Cadalbert, R., Wall, J., Güntert, P., Böckmann, A., Glockshuber, R., and Meier, B. H. (2015). Atomic-Resolution Three-Dimensional Structure of Amyloid β Fibrils Bearing the Osaka Mutation. *Angew Chem Int Ed Engl* 54, 331–335. doi:10.1002/anie.201408598.

Wasmer, C., Lange, A., van Melckebeke, H., Siemer, A. B., Riek, R., and Meier, B. H. (2008). Amyloid fibrils of the HET-s(218-289) prion form a beta solenoid with a triangular hydrophobic core. *Science* 319, 1523–1526. doi:10.1126/science.1151839.
